# Supplementary material for: “I’m in the Bluesky Tonight”: Insights from a year worth of social data
Source: PLoS One. 2024 Nov 5;19(11):e0310330. doi: 10.1371/journal.pone.0310330 (PMC11537377; doi:10.1371/journal.pone.0310330)
Supplement: Attachment — Submitted filename: Bluesky Rebuttal.pdf [file pone.0310330.s001.pdf]

We would like to thank the editor and the reviewers for their time and valuable feedback. Their suggestions and comments have significantly contributed to enhancing the quality of our paper. Below, we provide a detailed, point-by-point response to each report.

Academic Editor.

Q. *1. Please ensure that your manuscript meets PLOS ONE's style requirements, including those for file naming. The PLOS ONE style templates can be found at*

https://journals.plos.org/plosone/s/file?id=wjVg/PLOSOne_formatting_sample_main_body.pdf and

https://journals.plos.org/plosone/s/file?id=ba62/PLOSOne_formatting_sample_title_authors_affiliations.pdf

A. We have reviewed the style requirements and ensured compliance.

Q. *2. In your Methods section, please include additional information about your dataset and ensure that you have included a statement specifying whether the collection and analysis method complied with the terms and conditions for the source of the data.*

A. We moved the Ethics statement to the Materials and Methods section. The statement now specifies that the collection and analysis methods comply with the platform's terms of service.

Q. *5. We note that Figure 1 in your submission contain copyrighted images. All PLOS content is published under the Creative Commons Attribution License (CC BY 4.0), which means that the manuscript, images, and Supporting Information files will be freely available online, and any third party is permitted to access, download, copy, distribute, and use these materials in any way, even commercially, with proper attribution. For more information, see our copyright guidelines: http://journals.plos.org/plosone/s/licenses-and-copyright.*

We require you to either (1) present written permission from the copyright holder to publish these figures specifically under the CC BY 4.0 license, or (2) remove the figures from your submission

A. We updated the figure removing potentially copyrighted material. Moreover, the caption now clarifies the image source (screenshot taken by the author from their own device).

Q. 3-4.

These changes were addressed in the cover letter as requested.

Reviewer 1.

Q. *The "relational" data and the posts might not be aligned? I will try to explain better. If I understand correctly, in the first phase, the relational data (followers of various accounts) are collected, and subsequently, all the posts published on the timelines of the collected users. Potentially, some follower/followee relationships might be added or removed during the collection of the posts. I would ask the authors to discuss whether this might represent a limitation in some studies and how to manage it (e.g., if it were a problem, to have "relational" data and posts aligned, one might consider only the posts published up until the start of data collection for the followers?)*

A. As the reviewer notes, the “photograph” we take of a social media platform is not instantaneous but gradual, requiring time to be fully captured. Consequently, changes such as relation/post addition/removal may be overlooked if they happen after the corresponding area of the network is parsed. Thus, considering that a degree of information loss is inevitable at such a scale, we took some measures to limit its repercussions. Specifically, we last parsed the follower network on March 19th (this process started and ended on the same day), and released posts until March 18th (i.e., immediately before starting to parse follower relations). By doing so, we ensure that the misalignment is kept to a minimum (about 24 hours). We clarified this aspect in lines 135-139 by highlighting the advantage of this filtering process.

Q. *Text from row 98 to 101: I would like to know if the authors have encountered the same problem for replies. In other words, how did they separate replies from reposts? Does the metadata provided by Bluesky already provide this information?*

A. Bluesky references the post to which a reply is linked within post metadata. As the reviewer noted, we did not specify this aspect in the previous version of the manuscript. In the attached version, we state that replies and quotes have this relevant information as metadata, as opposed to reposts (lines 99-101).

Reviewer 2.

Q. *in line 43, the authors missed an upper case after a dot*

A. The typo is now fixed

Q. *I would rename the "Results" section to something like "Data analysis"*

A. The Results section was renamed to Data Analysis
